# Study on the clinical efficacy, safety and compliance of quality nursing intervention in the treatment of chronic heart failure combined with respiratory tract infections

**DOI:** 10.12669/pjms.40.7.7873

**Published:** 2024-08

**Authors:** Yinan Jia, Na Li, Xinxin Zhang, Zhiwei Lu, Xiaonan You, Qiaoling Xu

**Affiliations:** 1 Yinan Jia, Department of Internal Medical-Cardiovascular, Baoding NO.1 Central Hospital, Baoding, Hebei 071000, China; 2 Na Li, Department of Infectious Diseases, Baoding NO.1 Central Hospital, Baoding, Hebei 071000, China; 3 Xinxin Zhang, Department of Internal Medical-Cardiovascular, Baoding NO.1 Central Hospital, Baoding, Hebei 071000, China; 4 Zhiwei Lu, Department of Pulmonary and Critical Care Medicine, Baoding NO.1 Central Hospital, Baoding, Hebei 071000, China; 5 Xiaonan You, Department of Infectious Diseases, Baoding NO.1 Central Hospital, Baoding, Hebei 071000, China; 6 Qiaoling Xu, Department of Internal Medical-Cardiovascular, Baoding NO.1 Central Hospital, Baoding, Hebei 071000, China

**Keywords:** Quality nursing intervention, Chronic heart failure, Respiratory tract infections, Safety, Compliance

## Abstract

**Objective:**

To evaluate the clinical efficacy, safety and compliance of quality nursing intervention in the treatment of chronic heart failure combined with respiratory tract infections.

**Methods:**

This was a retrospective study. One hundred and twenty patients with chronic heart failure combined with respiratory tract infections were recruited at Baoding No.1 Central Hospital from June 2021 to March 2023 and randomly divided into the control group (n=60) and the experimental group (n=60). Patients in the control group were given regular specialist care on the basis of basic treatment, while those in the experimental group were given a quality care intervention model. The differences in clinical efficacy, improvement time of symptoms after treatment, etc. between the two groups were compared and analyzed.

**Results:**

The response rate of the experimental group was 88%, which was significantly higher than that of the control group (73%), with a statistically significant difference (P=0.04). The time of fever reduction, cough subsidence and lung rales disappearance in the experimental group were significantly shorter than those of the control group, with statistically significant differences (P<0.05). The incidence of nursing related adverse events in the experimental group was 8%, which was lower than that of 23% in the control group, with a statistically significant difference(P=0.03).

**Conclusion:**

Quality nursing intervention is an effective treatment for patients with chronic heart failure combined with respiratory infections, boasting a variety of benefits such as reduced nursing risk, improved quality of nursing, and increased patient compliance and satisfaction. It contributes to rapid symptom improvement and significant clinical efficacy.

## INTRODUCTION

Chronic heart failure is the end-stage manifestation of the cardiovascular system. It is a comprehensive disease caused by myocardial function and structure impairment due to myocardial infarction, valvular heart disease, coronary heart disease, cardiomyopathy and many other causes, which eventually lead to impaired systolic function and decreased left heart ejection.^1^ Its clinical manifestations include congestion of pulmonary circulation and systemic circulation, pulmonary moist rales, limited physical activity, dyspnea, edema, etc.^2^ Patients with chronic heart failure often suffer from respiratory dysfunction and respiratory tract infections during hospitalization due to pulmonary circulation congestion and decreased lung function.^3^ Among them, respiratory tract infections are also the most common trigger for heart failure or aggravation of heart failure symptoms.^4^

Chronic heart failure combined with respiratory tract infections is a great threat to patients, which can only be treated by symptomatic treatment modalities, such as improving cardiac function and lung function, controlling respiratory infections, and improving body immunity.^5^ the biggest obstacle faced by patients with chronic heart failure is the limitation of physical activity and cardiac insufficiency. Patients with limited physical activity are often in a state of despair, anxiety, guilt and other psychological disorders, with reduced compliance to treatment due to negative emotions. Therefore, simple symptomatic treatment is generally not very effective in improving lung and heart function of patients.^6^ Quality nursing intervention is a new type of nursing care that is tailored to a particular disease and delivered in a predetermined manner.^7^ A growing body of evidence^8^ shows that quality nursing intervention boasts improved quality of life for people with chronic diseases and reduced use of health services. In this study, quality nursing intervention was applied to treat patients with chronic heart failure combined with respiratory tract infections, to evaluate the clinical efficacy, safety and compliance of quality nursing intervention in the treatment of chronic heart failure combined with respiratory tract infections.

## METHODS

This was a retrospective study. One hundred and twenty patients with chronic heart failure complicated with respiratory tract infections admitted to the Baoding No.1 Central Hospital were recruited from June 2021 to March 2023 and randomly divided into two groups: the control group and the experimental group, with 60 cases in each group. No statistically significant differences were observed in the comparison of general information between the two groups, which were comparable (Table-I).

**Table-I:** Comparative analysis of general data of the experimental group and the control group (*χ̅*±*S*) n=60.

Index	Experimental group	Control group	t/χ^2^	P
Age (years)	64.51±9.54	65.03±8.46	0.32	0.75
Male (cases, %)	36(62%)	35(63%)	0.03	0.85
Medical history (years)	4.43±0.82	4.27±0.64	1.19	0.24
BMI (kg/m^2^)	22.49±5.03	22.62±5.21	0.14	0.89
***Cardiac function grading (NYHA)***				
Grade-I	15	17	0.17	0.68
Grade-II	42	38	0.60	0.44
Grade-III	3	5	0.54	0.46

P>0.05

### Ethical Approval

The study was approved by the Institutional Ethics Committee of Baoding No.1 Central Hospital on November 03, 2022(No.: [2022]047), and written informed consent was obtained from all participants.

### Inclusion criteria:

- Patients meeting the diagnostic criteria for chronic heart failure;^9^
- Aged 55-75 years old;
- Patients with symptoms of respiratory tract infections such as cough, fever and sputum;
- Patients with chest X-ray examination showing patchy and faint lung shadow and thickened texture;
- Patients with positive sputum culture and elevated white blood cells by a routine blood test;
- Patients who were conscious, without mental disorders, and able to actively cooperate with the implementation of treatment and nursing programs;
- Patients with informed consent and voluntary participation;
- Patients with complete clinical data;
- Patients who were able to cooperate with the completion of the study and had good treatment compliance.

### Exclusion criteria:

- Patients with a malignant tumor, severe primary disease, or cardiogenic shock;
- Patients with other vital organs such as liver and kidney dysfunction;
- Patients with a severe mental disorder or cognitive dysfunction;
- Patients with poor treatment compliance and inability to cooperate with treatment or care.

Patients in the control group were given routine specialist care, including lectures by the nurse in charge to the patients and their families on disease conditions, treatment and care points, precautions, etc.; nutritional diet guidance, encouragement of vitamin and high protein intake, reduction of fat and salt intake, as well as abstinence from smoking and alcohol; reasonable arrangements for rest time for the patients; necessary psychological counseling and comfort; and ECG monitoring for close monitoring of patients’ vital signs and condition changes. Patients with poor cardiac function were placed in a semi-recumbent position, with the head of the bed elevated, and given respiratory care and nebulized inhalation.

### Patients in the experimental group were given a quality nursing intervention model

including respiratory infection and chronic heart failure nursing. The specific methods are as follows. For respiratory tract infection nursing:

Psychological nursing: relevant reassurance work was done to relieve the patients’ tension and fear.

### Health education

the prevention of respiratory tract infections, and the method and purpose of aerosol inhalation were explained to the patients and their families to make them clear the purpose, operation method and precautions of aerosol inhalation and to improve the safety of treatment.

### Respiratory tract examination

Sputum aspiration was performed in time in case of high sputum; the aerosol inhalation volume was strictly controlled from small to large to avoid airway mucosal damage and airway spasm caused by sudden excessive aerosol inhalation volume; patients were closely observed for any discomfort performance during treatment.

### After aerosol inhalation

Patients were instructed to expel sputum and remove residual drugs around the mouth and nose; patients were allowed to go out after being observed for five minutes to confirm that there were no adverse reactions; patients and bedside family members were instructed on diet and nutrition to enhance patients’ immunity; nursing staff were instructed to do a good job in cleaning the room and to avoid patients’ contact with allergy-prone substances, such as feathers, flakes, dust, etc. For chronic heart failure nursing:

Patients were assessed for dyspnea, cough, sputum, hemoptysis, edema, etc., and instructed to self-monitor changes in symptoms and to notify the physician or nurse of any discomfort in a timely manner.

### Toxic reaction assessment of digitalis

Patients were assessed for nausea, vomiting, loss of appetite of gastrointestinal reactions and headache, dizziness, visual changes of neurological reactions, etc., and were given timely and appropriate treatment.

### Health behaviors

Including medication compliance, family rehabilitation training, sleep and diet. Patients were assessed for compliance with medical advice to take diuretics, digitalis, and other medications that reduce cardiac load and strengthen myocardial contractility. Patients with good compliance were praised, while those with poor compliance were given education and guidance, and their compliance was monitored together with their families.

Based on cardiac function, patients were assessed for aerobic exercise according to the exercise prescription given by the physician, and were instructed to eat low-salt, low-fat, vitamin-containing, light and easily digestible foods, to avoid a high cholesterol and high fat diet, and to stop smoking and drinking.

### Psychosocial aspects

Communication with patients and their families was carried out to clarify the psychological problems encountered by patients during hospitalization, such as despair, anxiety, guilt, etc. Psychological counseling was given in a timely manner when emotional abnormalities were detected.

### Observation indexes:

### Clinical efficacy

The clinical effect of the two groups before and after treatment was compared and analyzed. Marked response: cardiac function improved ≥ Grade-2, respiratory tract infection symptoms disappeared completely; Moderate response: cardiac function improved ≥ grade 1, respiratory tract infection symptoms partially disappeared; No response: No improvement or aggravation of cardiac function and respiratory tract infections. Overall response rate = (number of marked response cases + number of moderate response cases)/total number of cases × 100%.^10^

The improvement time of symptoms after treatment was compared between the two groups, including the time of fever reduction, cough subsidence, disappearance of lung rales, leukocyte recovery and sputum culture turning negative.

Comparative analysis of the incidence of nursing related adverse events: adverse events due to nursing staff’s non-standard operation or insufficient protective measures during hospitalization

### Comparative analysis of compliance

Complete compliance: patients can actively cooperate with treatment, follow medical advice and take medication on time, etc.; Partial compliance: patients can follow medical advice and take medication on time under the supervision of nursing staff or family members, with an average degree of cooperation; Non-compliance: patients cannot follow medical advice to take medication on time, or even interrupt treatment without authorization, with a very low degree of cooperation. Treatment compliance rate =(number of complete compliance cases + number of partial compliance cases)/total number of cases × 100%;^11^

The Patient Satisfaction Questionnaire Short Form (PSQ-18)^12^ was used to compare and analyze the patient satisfaction before and after the intervention, including very satisfied, relatively satisfied, satisfied, uncertain and dissatisfied. Total satisfaction = (very satisfied + relatively satisfied + satisfied)/total number of cases x 100%.

### Statistical analysis

All data in this study were statistically analyzed by SPSS 20.0 software, and measurement data were expressed as(*χ̅*±*S*). The power of test / confidence interval is 95%. Two independent sample t test was used for comparison between groups, paired t test was used to analyze data within groups, and χ^2^ test was used for the comparison of rates. P<0.05 indicates a statistically significant difference.

## RESULTS

The comparative analysis of clinical efficacy showed that the response rate of the experimental group was 88%, which was significantly higher than 73% of the control group, with a statistically significant difference (P=0.04) (Table-II).

**Table-II:** Comparative analysis of clinical efficacy of the two groups (*χ̅*±*S*) n=60.

Group	Marked response	Moderate response	No response	Overall response rate
Experimental group	20	33	7	53 (88%)
Control group	17	27	16	44 (73%)
*χ* ^2^				4.36
*P*				0.04

P<0.05.

After treatment and quality nursing intervention, the time of fever reduction, cough subsidence and lung rales disappearance in the experimental group were significantly shorter than those of the control group, with statistically significant differences (P<0.05). However, no statistically significant difference was observed in the time of leukocyte recovery and sputum culture turning negative between the two groups (P>0.05) (Table-III). The incidence of nursing related adverse events in the experimental group was 8%, which was lower than that of 23% in the control group, with a statistically significant difference (P=0.03) (Table-IV).

**Table-III:** Comparative analysis of symptom improvement time (d) between the two groups after treatment (*χ̅*±*S*) n=60.

Group	Fever reduction time*	Cough subsidence time*	Lung rales disappearance time*	Leukocyte recovery time	Sputum culture turning negative time
Experimental group	2.43±0.62	7.40±1.22	5.87±1.62	4.47±0.97	5.63±1.32
Control group	1.64±0.35	9.13±2.01	6.84±1.73	4.75±1.03	5.85±1.46
t	8.60	2.40	3.17	1.53	0.87
P	0.00	0.02	0.00	0.13	0.38

* P<0.05.

**Table-IV:** Comparative analysis of the incidence of nursing related adverse events between the two groups (*χ̅*±*S*) n=60.

Group	Phlebitis	Tube dislodgement	Infusion reaction	Pressure ulcer	Fall	Incidence*
Experimental group	1	0	4	0	0	5 (8%)
Control group	3	3	4	2	1	13 (22%)
*χ* ^2^						4.18
*P*						0.03

* P<0.05.

The compliance in the experimental group was 98%, which was significantly higher than 83% in the control group, with a statistically significant difference (P=0.00) (Table-V). The patient satisfaction in the experimental group was 95%, which was significantly higher than that in the control group (80%), with a statistically significant difference (P=0.01) (Table-VI).

**Table-V:** Comparative analysis of treatment compliance between the two groups after the intervention (*χ̅*±*S*) n=60.

Group	Complete compliance	Partial compliance	Non-compliance	Compliance rate*
Experimental group	48	11	1	59 (98%)
Control group	36	14	10	50 (83%)
χ^2^				10.44
P				0.00

* P<0.05.

**Table-VI:** Comparative analysis of patient satisfaction between the two groups (*χ̅*±*S*) n=60.

Group	Very satisfied	Relatively satisfied	Satisfied	Uncertain	Dissatisfied	Total satisfaction*
Experimental group	33	11	13	2	1	57 (95%)
Control group	25	14	9	5	7	48 (80%)
*χ* ^2^						4.22
*P*						0.01

* P<0.05.

## DISCUSSION

Our results showed that after quality nursing intervention, the response rate of the experimental group was 88% and that of the control group was 73%, with a statistically significant difference (P=0.04). The time of fever reduction, cough subsidence and lung rales disappearance in the experimental group were significantly shorter than those of the control group, with statistically significant differences (P<0.05), demonstrating the good effect of quality nursing intervention on patients’ recovery and improvement of symptoms.

Patients’ understanding of diseases and treatment methods is beneficial for their better participation in nursing care, and can also improve the prevention effect of diseases and avoid negative factors in a more rational way.^13^ Moreover, a more detailed explanation of the disease and the development of trust with the patients is a great aid to their understanding of the treatment process. In this way, nursing staff can urge patients to actively cooperate with treatment and nursing under the full trust and support of patients, which increases the success rate of treatment while reducing the occurrence of nursing-related adverse events. It was shown in our study that the incidence of nurse-related adverse events after nursing was 8% in the experimental group versus 23% in the control group(P=0.03). The compliance rate was 98% in the experimental group and 83% in the control group(P=0.00). Moreover, patient satisfaction in the experimental group was 95%, compared with 80% in the control group(P=0.01). This suggested the positive effect of quality nursing measures on alleviating negative emotions, while measures such as health guidance strengthened patients’ cognition and understanding of the disease and improve their awareness of self-care. In addition, quality nursing measures contributed to the change of patients’ bad behavioral habits, improved patient satisfaction and compliance, and accelerated their recovery.

Respiratory tract infections may cause the production of large amounts of catecholamines in the body’s immune system, which increases the cardiac load, induce abnormal myocardial contraction, and aggravate heart failure.^14^ It has been suggested^15^ that inflammatory response after respiratory tract infections can lead to pulmonary hypertension and reduced coronary blood supply. Jung et al.^16^ has demonstrated a positive correlation between respiratory tract infections and low physical activity in patients with heart failure. It can thus be seen that aggressive treatment of respiratory tract infections is a priority in the prevention of heart failure.^17^

Patients with heart failure combined with respiratory tract infections often suffer from high levels of mental pressure and psychological disorders. Such psychological and spiritual pressure will induce the activation of the body’s sympathy-adrenaline system, which will aggravate the left heart burden of patients, affect the immune status and the effectiveness of treatment,^18^ and cause adverse effects on subsequent treatment. It has been confirmed^19^ that patients with heart failure combined with respiratory tract infections are affected indirectly or directly by negative emotional interference in the treatment process, leading to a series of issues such as poor treatment outcomes and poor prognosis.

Quality nursing services aim to strengthen basic nursing with patients as the center, comprehensively implement nursing responsibility system, deepen nursing professional connotation, and improve the overall level of nursing services.^20^ “Patient-centered” means thinking for patients in terms of ideology and medical behavior and putting patients first in all nursing activities. Specifically, the needs of patients should be closely centered in the nursing process in order to improve the quality of service, control the cost of service, develop convenient measures, and simplify the work flow, with a view to providing patients with “high quality, high efficiency, low consumption, satisfaction, rest assured” medical services. As for the connotation of quality nursing service, it is to meet the basic needs of patients, ensure the safety of patients, maintain the physical comfort of patients, assist in balancing the psychology of patients, and obtain the coordination and support of patients’ families and society. In short, quality nursing should be used to improve patient and community satisfaction.^21^

### Limitations

Nevertheless, shortcomings are still visible in this study: small sample size and insufficient follow-up content. In response to this, more samples will be included and follow-up will be increased and extended in future clinical studies, in order to more objectively evaluate the advantages and disadvantages of the intervention and to benefit more patients.

## CONCLUSION

Quality nursing intervention is an effective treatment for patients with chronic heart failure combined with respiratory infections, boasting a variety of benefits such as rapid symptom improvement, significant clinical efficacy, reduced nursing risks, and improved patient compliance. It contributes to an overall improvement in the quality of nursing and satisfaction, which is worth promoting and applying in clinical practice.

### Authors’ Contributions:

**YJ and NL** carried out the studies, participated in collecting data, and drafted the manuscript, are responsible and accountable for the accuracy or integrity of the work.

**XZ and ZL** performed the statistical analysis and participated in its design.

**XY and QX** participated in acquisition, analysis, or interpretation of data and draft the manuscript. All authors read and approved the final manuscript.
